# The effect of Astym^®^ Therapy on muscle strength: a blinded, randomized, clinically controlled trial

**DOI:** 10.1186/s12891-015-0778-9

**Published:** 2015-10-29

**Authors:** Benjamin R. Kivlan, Christopher R. Carcia, F. Richard Clemente, Amy L. Phelps, RobRoy L. Martin

**Affiliations:** Department of Physical Therapy, John G. Rangos Sr.,School of Health Sciences, Duquesne University, Pittsburgh, PA 15282 USA; Tri-State Physical Therapy, Pittsburgh, PA USA; Palumbo Donahue School of Business, Duquesne University, Pittsburgh, PA USA; UPMC Center for Sports Medicine, Pittsburgh, PA USA

## Abstract

**Background:**

Astym^®^ therapy is a manual therapy intervention used to stimulate tissue healing, decrease pain, improve mobility, and improve muscle performance associated with musculoskeletal pathology. The purpose of this study was to determine if Astym therapy administered to the lower extremity would result in an immediate change of maximal force output during a unilateral isometric squat test among individuals with a lower extremity injury.

**Methods:**

Forty-five subjects (14 males; 31females) between 18 and 65 years of age were randomized into 3 treatment groups: 1) Control group – received no treatment 2) Placebo group – received a sham Astym treatment 3) Astym therapy group– received Astym therapy to the lower extremity. A baseline measure of maximal force output (pre-test) during a unilateral isometric squat was performed. The subjects then received the designated treatment intervention. Immediately following the treatment intervention, maximal force output (post-test) was retested using identical testing procedures by an investigator who was blinded to the treatment intervention received by the subjects. The percent change of maximal force output from pre-test to post-test measures was compared using a one-way analysis of variance. A Tukey’s post-hoc analysis determined the statistical differences between the groups.

**Results:**

The treatment intervention had a significant effect on the percent change of maximal force output [F(2,42) = 7.91, *p* = 0.001]. Tukey’s post hoc analysis demonstrated that the percent change of maximal force output was significantly greater in the Astym group (15 ± 18 % change of Newtons) compared to the placebo (−6 ± 11 % change of Newtons; *p* = 0.0001) and control (−1 ± 17 % change of Newtons; *p* = 0.0014) groups. No significant difference (*p* = 0.68) was noted between the control and placebo groups.

**Conclusions:**

Astym therapy to the involved lower extremity increased maximum force output during an isometric squat test immediately following treatment. The results of this study suggest that Astym therapy can immediately improve muscle performance (maximal force output) for patients presenting with muscular weakness caused by a lower extremity musculoskeletal injury.

**Trial registration:**

Clinicaltrials.gov NCT02349230. Registered 23 January 2015.

## Background

Astym^®^ therapy (Performance Dynamics, Muncie, Indiana, USA) is a manual therapy technique used to facilitate soft-tissue healing and address the impairments associated with common musculoskeletal pathology [1]. Astym therapy is applied with specialized instruments that have shown evidence of stimulating soft-tissue healing [2, 3], and improving impairments such as pain, limitations in mobility, and muscle weakness that may accompany musculoskeletal pathology [4–13]. Anecdotally, therapists have noted Astym therapy can invoke an immediate improvement in muscle performance. However, this effect has yet to be studied in a clinically controlled trial.

Two controlled clinical trials [1, 14] and several case studies have shown Astym therapy can reduce pain and improve function when used to treat common musculoskeletal pathologies including epicondylosis, [1,6] carpal tunnel syndrome, [15] Achilles tendinopathy, [9] hamstring tendinopathy, [10] and patellar tendinopathy. [14] Astym therapy has also been successfully used to improve joint mobility as a result of excessive soft-tissue scarring and fibrosis [5, 7, 8, 12]. In two separate case studies, the addition of Astym therapy to the treatment plan resulted in clinically significant changes to knee joint range of motion when previous conservative and surgical interventions had failed [7, 8]. Astym therapy was also used successfully to restore the range of motion to pre-injury levels in 2 separate cases of individuals with ankle joint dysfunction caused by excessive fibrosis [12, 13].

The effects of Astym therapy on muscle performance are not so clear. Muscle performance can be defined as the combination of the strength, power, and endurance of a muscle or group of muscles necessary to execute a specific task or functional activity [16]. Muscular strength is a component of muscle performance, defined as the amount of maximal volitional force produced by the contraction of a single muscle or a group of muscles [17]. Lower extremity muscular strength that is measured in the closed kinetic chain (ex. squat or leg press), has been associated with an individual’s ability to walk and negotiate stairs [18]. A deficit of lower extremity muscular strength has been shown to be a risk factor for falls in an elderly population [19]. In a younger, active population, squat strength is associated with athletic performance measures of sprinting, [20]^,^ vertical jump height, [21] and agility test time [21]. Based on the literature cited above, there is evidence to suggest that lower extremity muscular strength is an important component of muscle performance for a wide variety of functional activities. Exploring the effect of Astym therapy on force output could impact how clinicians implement a treatment program to improve muscular strength and improve lower extremity function.

Currently, there is no evidence to substantiate anecdotal reports that Astym therapy improves muscle performance. There is also a need to further understand how Astym therapy may be used to improve muscle performance in patients with musculoskeletal injuries. The purpose of this study was to determine if Astym therapy administered to the lower extremity would result in an immediate change of maximal force output during a unilateral isometric squat test among individuals with a lower extremity injury. The hypothesis was that subjects that received Astym therapy would have a significant improvement of maximal force output when compared to a group that received no treatment or a group that received sham Astym treatment.

## Methods

### Experimental design

A double-blinded, repeated measures design was used to investigate the effect of Astym therapy on muscle performance of the lower extremity. The dependent variable was the maximal force generated during a unilateral isometric squat test. The independent variable was the treatment received by the subjects: 1) Astym therapy - received a lower extremity Astym therapy2) Control-received no treatment; 3) Placebo-received a sham Astym therapy. Subjects were randomly assigned to receive the control, placebo, or Astym therapy intervention and were blinded to the treatment of their assigned group. The primary investigator (BRK) performed the control, placebo, or Astym therapy interventions. A second investigator (LB), blinded to the treatment, administered the pre- and post-treatment isometric squat tests. The primary investigator (BRK) who performed the data analysis remained blinded to the results of the isometric squat tests until the post-treatment tests were completed for all subjects.

### Subjects

A total of 45 subjects between the ages of 18 to 65 years that met the inclusion/exclusion criteria were recruited from an outpatient physical therapy center. Sample size estimates were projected based on data from a pilot study. Using the exact data collection procedures described in this manuscript, the percent change of maximum force output was collected for 12 volunteers that received the control treatment, 12 volunteers that received the placebo treatment, and 12 volunteers that received the Astym therapy. The data were used to determine the mean difference and the effect size of the control and placebo groups to the treatment group. A commercially available power analysis software program (JMP Pro 10; Cary, North Carolina) was used to calculate the sample size needed to obtain 80 % power with alpha set at 0.05 based on the smallest effect size (Astym-Control) determined from the pilot study data. The results of the power analysis concluded that a sample size of 15 subjects per group was needed to detect a minimal difference of 14 % between the groups. Potential subjects were recruited during an initial outpatient physical therapy visit and continued until each group had 15 subjects.

Inclusion criteria for subjects were: 1) males or females aged between 18 and 65 years, 2) a referral from a medical doctor for physical therapy services for a musculoskeletal injury/condition to the lower extremity, and 3) unilateral symptoms to the lower extremities. Exclusion criteria included: 1) medical history of hemophilia or other blood coagulation disorders; 2) medical history of cardiovascular disease including those with previous cardiovascular surgery and uncontrolled hypertension; 3) current use of prescription blood thinners; 4) a history of metastatic disease; 5) neuropathy of the lower extremity; 6) current complaints of lumbar or shoulder symptoms; 7) an active infection (or taking medication for an infection) 8) conditions affecting both lower extremities and 9) previous treatment that included Astym therapy. All subjects were asked to read and sign an informed consent form approved by the Duquesne University Institutional Review Board and to complete the Lower Extremity Functional Scale to objectify functional limitations caused by their condition. Once enrolled, subjects were excluded if they were functioning at an extreme high level. A functional deficit was operationally defined as Lower Extremity Functional Scale score that was greater than the minimal clinically important difference (9 points) that has been previously established in the literature [22]. Therefore, subjects that scored greater than a 70 out of a possible 80 points on the Lower Extremity Functional Scale were excluded from the study.

### Data collection

**Fig. 1:**
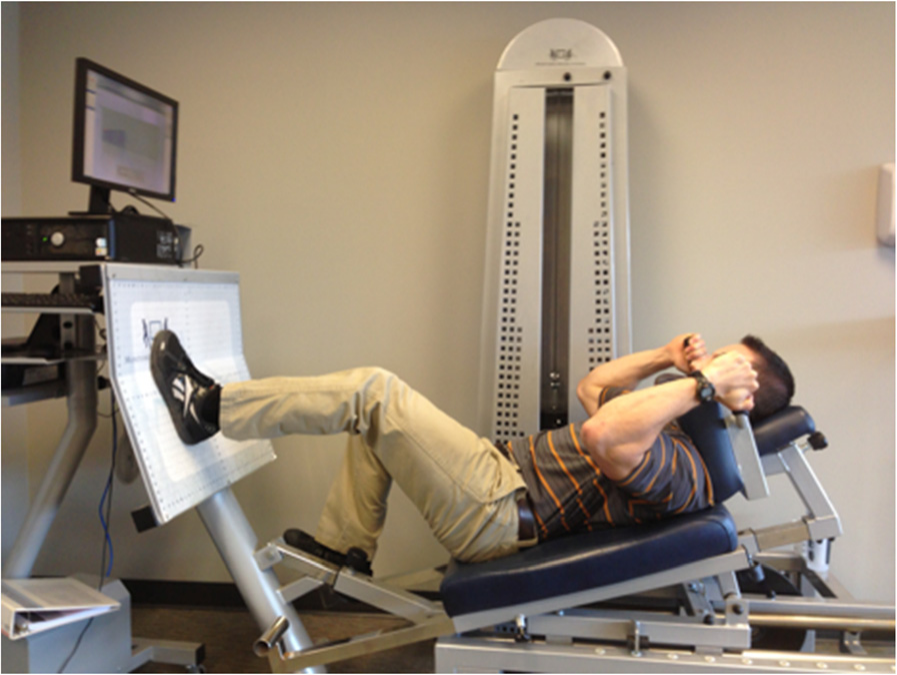
Maximal isometric squat test. Patient performing a maximal isometric squat test on the monitored rehab systems^®^ computerized leg press machine

Maximum force output during a unilateral isometric squat test was measured using a computerized leg press machine (Fig. 1) equipped with a load cell (CDM Sport; Fort Worth, TX). The load cell was tested by the manufacturer and demonstrated less than 0.02 % error for repeatability, zero balance, creep, non-linearity and hysteresis [23]. Data from a pilot study demonstrated excellent criterion validity for the computerized leg press machine to a digital force dynamometer with a Pearson correlation coefficient of 0.99. The analysis determined the typical error of the estimate to be 10.69 Newtons (95 % CI: 8.13–15.62 Newtons) and test-retest reliability of 0.99 (Appendix A). The standard error of the measurement was determined to be 2.7 % change with a minimal detectable change of 7.5 % change.

All data collection procedures were identical for each subject. Demographic information collected included age, height, weight, gender, lower extremity-dominance, and musculoskeletal diagnosis as determined by assimilation of a physician prescription and office notes, current subjective complaints/symptoms, and objective findings from physical therapy examination. Subjects filled out a medical history form that included items specific to the exclusion criteria. The subjects also filled out a self-reported functional questionnaire containing the numeric pain scale (0–10) and the Lower Extremity Functional Scale. Once the subject completed the forms, they were asked to ‘warm-up’ by cycling at a self-selected pace on a lower body ergometer (Sports Art c530 Lower Body Ergometer; Woodinville, WA) for 5 min.

Next, maximum isometric force during a squat test was measured for each lower extremity using a computerized leg press machine (CDM Sport; Fort Worth, TX). The lower extremity tested first was randomly selected for each subject by a coin flip. The leg press was adjusted for the designated lower extremity such that the subject’s knee joint was placed and maintained at 70° of knee flexion as determined by a standard 8-in. goniometer (AliMed 5055 - Med. International Standard 8-in. Goniometer, Dedham, MA). Measurement of knee flexion angle was done with the fulcrum of the goniometer placed over the lateral epicondyle of the knee, the stationary arm in alignment with the greater trochanter, and the mobile arm in alignment with the lateral malleolus. A grid system on the footplate of the leg press was used to standardize the foot position and to ensure that the foot, ankle, and hip joints were in alignment in the sagittal plane and the crest of the tibia was parallel to the floor. A testing protocol described by Carcia et al. [23] was utilized to collect maximum force output during the unilateral isometric squat test. Each subject was given a consistent, pre-scripted instruction to push through their heel against the footplate of the leg press a total of five times for 3 s each. The first repetition was performed at approximately 50 % effort, the second at 75 % effort, and the remaining three repetitions at 100 % effort. No verbal or visual feedback on performance was given during the testing. The average of the maximum force output (Newtons) produced during the final three trials was used to represent the subject’s maximal force output during the unilateral isometric squat test. The protocol accounted for possible learning/fatigue effects that may have occurred during repeated testing and was validated in a pilot study performed prior to the initiation of this research study utilizing a 3 s:30 s work/rest ratio over10 consecutive trials on the same lower extremity [24]. Pain was monitored before and after isometric testing using the numeric pain scale. Once the testing had been completed on the designated lower extremity, the opposite lower extremity was tested using the same testing procedures to determine a side-to-side comparison of strength. In healthy adults, a 2.8 % and 5 % side-to-side difference in unilateral squat strength is considered normal [25]. For the purpose of this study we operationally defined a strength deficit as a 10 % deficit of the involved side compared to the uninvolved side. Subjects that had less than a 10 % deficit, were not considered to have a significant strength deficit caused by their musculoskeletal pathology and were dismissed from the study.

**Fig. 2:**
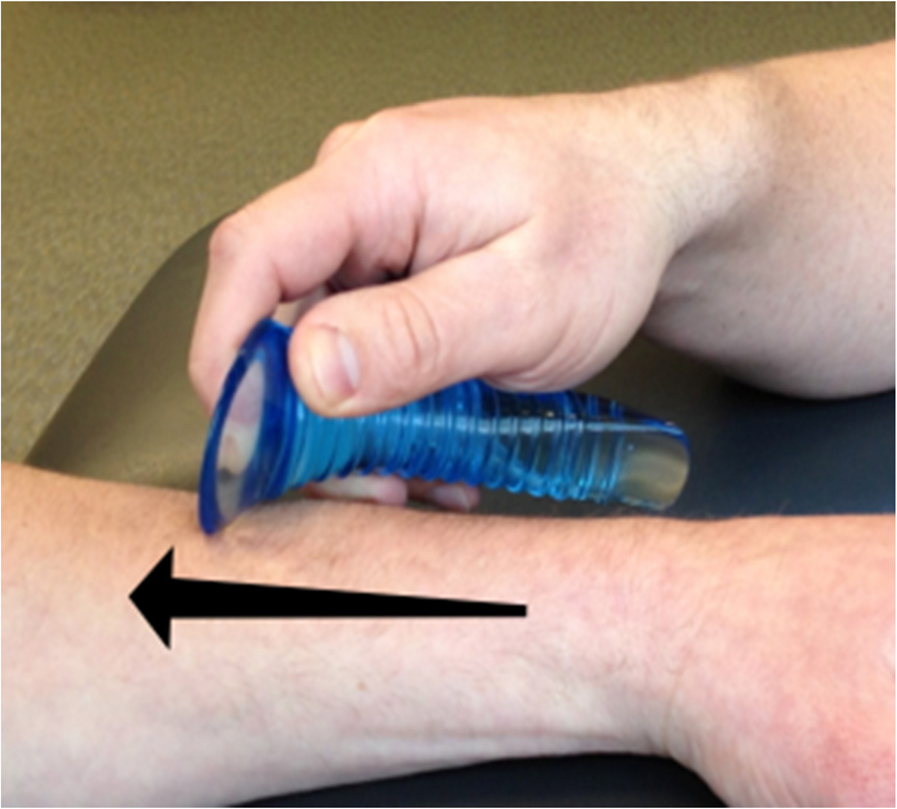
Astym therapy. A therapist performs Astym therapy with the Astym instruments

Next the subjects were randomly assigned to the control, placebo, or treatment group. Random assignment to the groups was determined using a random numbers generator (http://www.graphpad.com/quickcalcs/randomize1/) to create three equal groups of 15 subjects. The treatment group received Astym therapy in a manner previously described by Sevier and Stegink-Jansen [1] to the muscles of the anterior and lateral compartments of the leg, the gastrocnemius/soleus muscle complex, the quadriceps muscle group, the hamstrings muscle group, the gluteus maximus, and the gluteus medius muscles on the involved side (Fig. 2). The investigator performing the Astym therapy (BRK) has been certified in this form of therapy and has over 4 years experience administering Astym therapy for lower extremity musculoskeletal dysfunction.

**Fig. 3:**
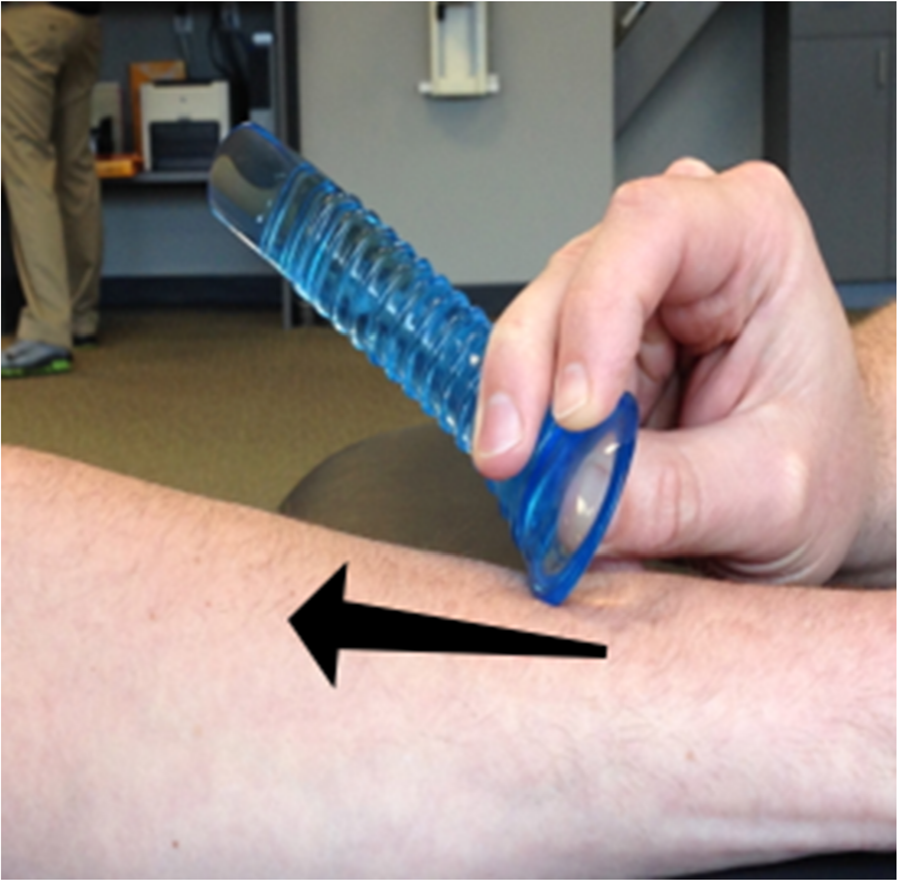
Sham Astym therapy. The non-treatment portion of the Astym instrument is glided lightly over the skin during the sham Astym treatment

The control group did not receive any treatment and was asked to sit on a treatment table for 12 min (the average time it took to perform an Astym therapy treatment in the pilot study). The placebo group received a sham Astym treatment. The sham treatment was analogous to an effleurage massage with the Astym instruments. The sham treatment was different from the real Astym therapy in that the non-treatment portion of the instrument was applied with very light pressure over the skin (Fig. 3).

Once the designated treatment intervention was completed, the subject was immediately retested on the computerized leg press machine using the identical testing procedures as described above. A second investigator (LB) blinded to the type of treatment the subject received administered all of the unilateral isometric squat tests. The investigator performing the Astym therapy did not have access to test results until testing was completed for each subject. Once the post-test was complete the subject satisfied the obligations of the research study and resumed the normal course of his/her care.

### Statistical analysis

All data was entered into SPSS Version 20 (SPSS Inc.; Chicago, IL) for statistical analysis. Descriptive statistics (means/standard deviations/range) of age, height, weight, self-reported functional score, pre-treatment pain rating, and post-treatment pain rating of the subjects was reported and compared between groups with an analysis of variance. The frequency of gender, the medical diagnoses by type (musculotendinous versus non-contractile), and region (proximal portion of the lower limb versus distal portion of the lower limb) for each respective treatment group was compared using a chi-square analysis. The percent change of maximum force output from pre-test to post-test was calculated by the following formula:

$$ \begin{array}{l}\underline {\mathrm{Post}\hbox{-} \mathrm{Test}\kern0.24em \hbox{-} \kern0.24em \mathrm{P}\mathrm{r}\mathrm{e}\hbox{-} \mathrm{Test}}\\ {}\kern1.44em \mathrm{P}\mathrm{r}\mathrm{e}\hbox{-} \mathrm{Test}\kern2.52em \mathrm{X}\kern0.24em 100=\mathrm{Percent}\;\mathrm{Change}\;\end{array} $$

The mean of the percent change for each group (Control, Placebo, Astym therapy) was compared using a one-way analysis of variance with a predetermined alpha set at 0.05. A Tukey’s post-hoc analysis was then used to determine which groups were statistically different from each other.

## Results

### Subjects

**Fig. 4:**
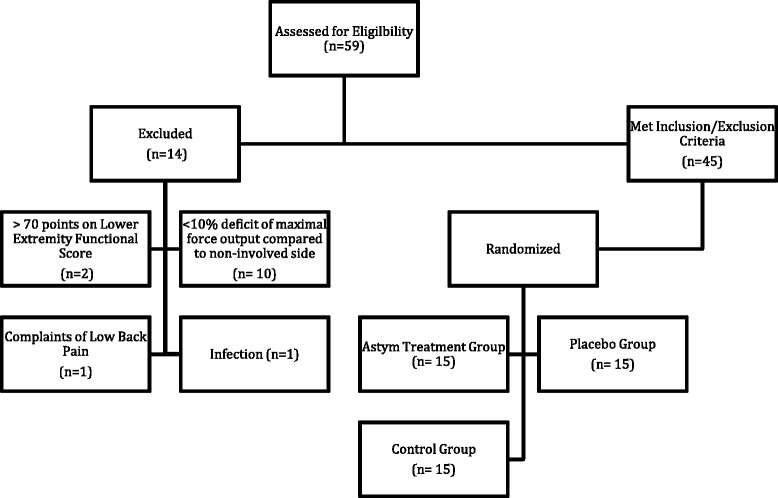
Flow diagram of subjects

A total of 59 subjects were enrolled in the study. A flow diagram of the subjects enrolled in the study is represented in Fig. 4. Ten subjects were excluded from the study because they did not exhibit a 10 % strength deficit of the involved side compared to the uninvolved side, 2 subjects scored greater than 70 points on the Lower Extremity Functional Score, 1 subject had a medical history of low back pain within the past 6 months, and 1 subject was taking medication for an infection.

**Fig. 5:**
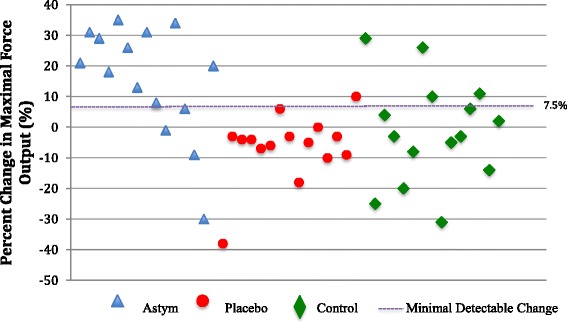
Plot graph of percent change in maximal force output according to treatment group. Astym group (blue triangle); placebo group (red circle) control group (green diamond); minimal detectable change (purple dotted line)

Data were collected on a total of 45 subjects. The average age, height, weight, self-reported functional score, pre-treatment pain rating, post-treatment pain rating, and involved side to uninvolved side strength deficit is reported according to each respective treatment group in Table 1. The analyses of variance demonstrated no statistical difference between the treatment groups for age (*p* = 0.19), height (*p* = 0.60), weight (*p* = 0.72), self-reported functional score (*p* = 0.99), pre-treatment pain rating (*p* = 0.85), post-treatment pain rating(*p* = 0.08), and involved side versus uninvolved side strength deficit (*p* = 0.56). The percentages of gender, lower extremity dominance, and involved side of the subjects are also organized according to treatment group in Table 1. A chi-square analysis demonstrated no significant difference in the female to male ratio (*p* = 0.48), lower extremity dominance ratio (*p* = 0.76), or involved side ratio (*p* = 0.77) for the subjects between the three treatment groups. Diagnoses were also not statistically different between treatment groups according to the region (distal or proximal; *p* = 0.71) and type (musculotendinous or non-contractile; *p* = 0.69) (Table 2).

**Table 1:** Characteristics of subjects according to treatment group

	Astym^®^ (mean ± SD)	Placebo (mean ± SD)	Control (mean ± SD)	TOTAL (mean ± SD)
Age (years)	42 ± 12	43 ± 13	35 ± 12	40 ± 13
Height (cm)	166 ± 13	168 ± 12	170 ± 9	168 ± 11
Weight (kg)	68 ± 11	70 ± 14	75 ± 20	71 ± 15
Functional score (0–80 points)	60 ± 10	60 ± 9	60 ± 8	60 ± 9
Pre-treatment pain rating 0–10)	2 ± 2	2 ± 2	3 ± 2	2 ± 2
Post-treatment Pain Rating (0–10)	2 ± 2	3 ± 2	3 ± 2	3 ± 2
Gender (% Female)	80 %	67 %	67 %	71 %
Lower extremity dominance (% Left)	7 %	13 %	7 %	9 %
Involved side (% Left)	40$	53 %	47 %	47 %

SD = standard deviation

**Table 2:** Frequency of diagnoses by region and type according to treatment group

	Astym	Placebo	Control	Total
Diagnosis by region				
Proximal total	10	12	11	33
Hip	2	0	2	4
Thigh	2	6	3	11
Knee	6	6	6	24
Distal total	5	3	4	12
Leg	0	1	0	1
Ankle	3	0	2	5
Foot	2	2	2	6
Diagnosis by type				
Musculotendinous	5	5	7	17
Non-contractile	10	10	8	28

### Percent change in maximal force output

A one-way analysis of variance demonstrated the treatment intervention had a significant effect on the percent change of maximal force output [F(2,42) = 7.91, *p* = 0.001] (Table 3). Tukey’s post hoc analysis demonstrated that the percent change of maximal force output was significantly greater in the Astym group (15 ± 18 % change of Newtons) compared to the placebo (−6 ± 11 % change of Newtons; p = 0.0001) and control (−1 ± 17 % change of Newtons; *p* = 0.0014) groups. No significant difference (*p* = 0.68) was noted between the control and placebo groups. Table 4 presents the mean and standard deviation of the percent change of maximum force output according to treatment group and Table 5 compares the mean differences and the level of significance (*p*-value) between each of the group comparisons.

**Table 3:** Summary table for analysis of variance for percent change in maximal force output (Newtons)

Source	*df*	*Sum of squares*	*Mean square*	*F*	*p*	η^*2*^
Between groups	2	3902.53	1951.27	7.91	0.001	0.27
Within groups	42	10366.28	246.82			
Total	44	14268.80

**Table 4:** Mean, standard deviation, and range of force output according to treatment group

Group	Pre-treatment force output (Newtons)			Post-treatment force output (Newtons)			Percent change in force output (%)		
	Mean	SD	Range	Mean	SD	Range	Mean	SD	Range
Astym	994	527	354-2465	1150	630	475-2909	15	18	−30 -35
Placebo	965	533	371-1936	918	515	350-1861	−6	11	−38 - 10
Control	1043	646	212-2672	972	503	234-2128	−1	17	−31 - 29

SD = Standard Deviation

**Table 5:** Tukey’s pairwise mean differences of group comparisons

Group comparison	Mean difference	Significance (p-value)
Astym versus placebo	21 %	0.001
Astym versus control	16 %	0.014
Control versus placebo	5 %	0.675

## Discussion

The purpose of this study was to determine if Astym therapy administered to the lower extremity would result in an acute change of maximal force output during a unilateral isometric squat test among subjects presenting with weakness associated with a musculoskeletal injury to the lower extremity. Subjects that received Astym therapy increased maximal force output of the lower extremity immediately following treatment by an average of 15 % from pre-treatment values. This was significantly greater (*p* < 0.01) than the average 1 % and 6 % decrease in maximal force output (Newtons) demonstrated in the control and placebo treatment groups, respectively. The placebo treatment and control treatment groups were found not to be statistically different (*p* = 0.68).

An ancillary analysis was performed to evaluate the individual performances of the subjects. Figure 5 shows the percent change in maximal force output of each of the subjects according to the respective treatment groups. Eleven out of the 15 subjects that received Astym therapy had an improvement of maximal force production greater than the minimal detectable change of 7.5 % established for the isometric squat test during a pilot study. The minimal detectable change represents an estimate of the smallest amount of change that is not due to measurement error and may be used to determine if the individual performances were likely due to measurement error or a true change in maximal force output [26]. Conversely, only 4 subjects that received the control treatment and 1 subject that received the placebo treatment exhibited a positive percent change in maximal force output greater than the minimal detectable change.

The plot graph of individual performances (Fig. 4) shows a wide dispersion of values within the Astym therapy group, which explains the variation in standard deviation of the percent change in maximal force output. Two of the four of subjects in the Astym therapy group that did not improve beyond the minimal detectable change had diagnoses involving the foot and ankle region. Further examination of the results of the Astym therapy group revealed that subjects that were diagnosed with a condition affecting the proximal aspect of the lower extremity (hip, thigh, and knee regions) tended to have a greater percent change in maximal force output compared to the subjects with diagnoses affecting the distal portion of the lower extremity (leg, ankle, and foot). Table 6 presents the average percent change in maximal force output according to the location of the subject’s musculoskeletal diagnosis.

**Table 6:** Percent change in maximum force output following astym^®^ treatment by diagnosis region and type

Diagnosis categories					
Region				Type	
Hip-thigh-knee regions		Leg-ankle-foot regions		Musculotendinous	Non-contractile
Hip	15 % (*n* = 2)	Leg	NA (*n* = 0)	21 % (*n* = 5)	13 % (*n* = 10)
Thigh	28 % (*n* = 2)	Ankle	17 % (*n* = 3)		
Knee	20 % (*n* = 6)	Foot	−11 % (*n* = 2)		
Total	20 % (*n* = 10)	Total	5 % (*n* = 5)		

The observation that those with foot and ankle related diagnoses did not respond as well as those with diagnoses related to the hip, thigh and knee could be related to the specific demands of the isometric squat test. Muscles of the hip, thigh, and knee regions have shown greater muscle activation during a squat compared to muscles of the leg, ankle, and foot regions [27]. Thus the isometric squat test may be more likely to have a positive change in maximal force production for individuals with a diagnosis affecting the proximal portion of the lower extremity. The current study was not powered to perform a statistical comparison that would reveal whether the percent change of maximal force output was indeed influenced by the location of the individual’s diagnosis, but may provide the groundwork for a future study that investigates the influence on the location of diagnosis on changes in muscle performance following Astym therapy.

The type of diagnoses may have also contributed to the variance in the percent change of maximal force output within the Astym therapy group. The subjects that participated in the study all had diagnoses affecting the musculoskeletal system. These diagnoses were further categorized by involvement of contractile (musculotendinous) and non-contractile structures. Table 6 shows the mean percent change of maximal force output according to diagnoses involving musculotendinous versus non-contractile structures. The subjects in the Astym therapy group that were diagnosed with a musculotendinous condition had an average percent change in maximal force output of 21 % versus 13 % for those with a diagnosis involving non-contractile structures. An accurate statistical comparison cannot be made with the small sample size from the current study. The findings do, however, illustrate the need to perform a future investigation to determine the effects of diagnosis type on muscle performance following Astym therapy.

### Comparison to Other Therapeutic Interventions

The subjects from the current study that were randomized into the Astym therapy group demonstrated an average increase in maximal force output of 15 %. This increase of muscular strength is comparable to 14-23 % increases in muscular strength that have been reported immediately following joint mobilization of the lower extremity [28–30]. The improvements of muscular strength also compare favorably to increases documented between 5.20 and 9.4 % of lower extremity muscle strength in response to a whole-body vibration stimulus [31–33]. However, the results of the current research project are in direct conflict with previous research documenting 0-11 % decreases in lower extremity muscular strength in response to various forms of massage [34–36]and soft tissue mobilization using devices such as “the Stick” [37] and foam rollers [36, 37]. This demonstrates the unique effect of Astym therapy to acutely improve muscular performance compared to these other forms of manual therapy. The results of this study indicate there is an immediate effect on muscle strength, however this is an initial study that did not assess chronic effects of Astym therapy as part of a complete rehabilitation program. Therefore further evidence is still needed in order to fully support the use of Astym in the management of muscle weakness following injury.

### Clinical significance

Multi-joint, lower extremity muscular strength is directly related to the functional abilities of an individual [38]. Muscular strength measured with a unilateral squat test has been associated with ambulatory and stair climbing function [18]. Lower extremity muscular weakness is also a risk factor for falls in an elderly population [19]. In a younger, athletic population, lower extremity strength has been related to sprinting speed as well as measures of agility and jumping ability [20, 21, 39]. The consensus of current scientific literature suggests multi-joint, lower extremity strength has implications to a wide range of functional activities, from basic ambulatory function to advanced athletic performance.

Maximizing the effectiveness and efficiency of treatment sessions in a physical therapy practice is important in today’s health care environment where a physical therapist may be challenged to manage a patient’s deficits in a limited number of visits. The results of the current study support the use of Astym therapy in the management of patients with a documented weakness from a musculoskeletal injury/condition. Therapists may plan to utilize Astym therapy before engaging the patient in strengthening exercises or performing functional tasks that are limited by the strength of the patient. However, further study is required to determine if the increase of strength would translate to improved performance of functional tasks such as stair climbing or athletic activities.

### Limitations

A limitation to the study is that the mechanisms through which Astym therapy affected muscle performance is unknown. From a review of the literature, we may speculate that pain modulation [40, 41], neuromuscular facilitation [42–44], increased blood flow [45–52], and increases of intracellular calcium within muscle tissue [53–58] are possible mechanisms by which Astym therapy may acutely increase muscle performance. It is also unknown if Astym therapy worked directly on the injured structure or simply enhanced the performance of the non-injured muscles that contributed to isometric squat performance. The duration of the observed effect of Astym therapy on muscle performance is also unknown. Further study is needed to explain the physiological mechanisms through which Astym therapy may affect muscle performance and the duration that this effect may last. Another limitation of the study is that all of the subjects enrolled in the current study presented with a measurable strength deficit of at least 10 % when compared to the non-involved side. Therefore it remains unknown how Astym therapy may influence strength in those without an apparent strength deficit. Anecdotal reports from athletes have noted enhanced athletic performance immediately following Astym therapy. The results of the current study are encouraging that Astym therapy may facilitate athletic performance by improving muscular strength, but the sample from this study included only subjects that had muscular weakness and a known injury. Further study is required to determine the effects of Astym therapy on athletic performance.

## Conclusions

Astym therapy to the involved lower extremity increased maximum force output during an isometric squat test immediately following treatment. Subjects with muscular weakness that receive Astym therapy may expect an average improvement of 15 % in muscular strength. Subjects that received the control and placebo treatment did not show an acute improvement in maximal force output. The results of this study suggest that Astym therapy can immediately improve muscle maximal force output for patients presenting with muscular weakness caused by a lower extremity musculoskeletal injury. Future research is needed to understand the physiologic mechanisms that explain how Astym therapy increases muscular strength, the longevity of the observed increases in muscular strength, and to determine if Astym therapy will also result in acute changes in muscle power, functional abilities, and athletic performance.

## Appendix A

Reliability of the computerized leg press machine. A pilot study was performed to establish test-retest reliability of the Computerized Leg Press Machine. Twelve subjects performed 3 repetitions of maximal isometric testing on the computerized leg press machine. After a 12 min rest, maximal isometric testing on the computerized leg press machine was repeated with an additional 3 repetitions. An intra-class correlation coefficient (ICC) was computed from the average of the first 3 repetitions and the average of the final 3 repetitions of maximal isometric testing. Test-retest reliability of maximal isometric testing using the computerized leg press machine was determined with an ICC(2,1) of 0.99. The standard error of the measurement was determined to be a 2.7 % change in maximal force output. Using a 95 % confidence interval, the minimal detectable change was determined to be 7.5 % change in maximal force output.
